# An Exploration and Technical Notes for Advanced Airway Management on the Ski Slope: A Simulation Experiment

**DOI:** 10.1155/2021/9241891

**Published:** 2021-12-06

**Authors:** Peng Bai, Tian Xia, Zhongwei Yang, Wei Huai, Xiangyang Guo, Fang Zhou

**Affiliations:** ^1^Department of Anesthesiology, Peking University Third Hospital, 49 North Garden Road, Haidian District, Beijing 100191, China; ^2^Department of Orthopaedics, Peking University Third Hospital, Engineering Research Center of Bone and Joint Precision Medicine, 49 North Garden Road, Haidian District, Beijing 100191, China; ^3^Trauma Center, Peking University Third Hospital, 49 North Garden Road, Haidian District, Beijing 100191, China; ^4^Department of Emergency, Peking University Third Hospital, 49 North Garden Road, Haidian District, Beijing 100191, China

## Abstract

**Background:**

Skiing is a high-risk winter sport, and the rate of injury fatality is the highest compared to other winter sports. During skiing rescue, the harsh natural environments will increase the difficulty of artificial airway establishment. There has been no research focusing on the establishment of the artificial airway during skiing rescue site. This study aims to simulate the real-world scenario, calculating and comparing the operation time of different artificial airways on the cold slope, and to explore the optimal method of establishing artificial airway on the cold slope, sharing our experience, technical notes, and pitfalls we encountered, hoping to help establish a standard operating procedure in advanced airway management on the ski slope.

**Methods:**

The simulated human was placed on the cold slope with the head under the feet. Artificial airway was established by the same anesthesiologist using endotracheal intubation (endotracheal intubation group), LMA Supreme laryngeal mask (LMA group), and I-gel laryngeal mask (I-gel group). Each method was repeated 5 times, and the operation time and whether it was successful by one attempt were recorded and compared between groups.

**Results:**

Three groups of artificial airway were successful by one attempt.. The bite block dropped and drifted away for one time in the endotracheal intubation group. Operation time is 209.2 ± 32.7 seconds in the endotracheal intubation group, 72.2 ± 3.1 seconds in the LMA group, and 52.6 ± 4.2 seconds in the I-gel group. ANOVA showed that there was a significant difference in the operation time among the three groups (*p* < 0.001). Tukey's post hoc test showed that there were statistically significant differences between the endotracheal intubation group and the other two groups in operation time, *p* < 0.001, while there was no significant difference between the LMA group and I-gel group (*p*=0.275).

**Conclusion:**

The artificial airway can be completed by endotracheal intubation and LMA and I-gel laryngeal mask insertion on the cold slope. Artificial airway with the I-gel laryngeal mask takes the shortest time in this study. Extra caution should be paid to slippery and drifting.

## 1. Background

Skiing is a high-speed and high-risk winter sport driven by gravity and slope, and the rate of injury fatality is the highest compared to other winter sports. A total of 369 deaths were recorded on Austrian ski slopes between 2008 and 2018 [1], and more than 19,000 out-of-hospital cardiac arrest cases were reported by the Northern French Alps Emergency Network from 2004 to 2014 [2]. High altitude, cold, and other environmental conditions may also increase the risk of cardiac arrest. Regardless of the need of respiratory support during the on-site skiing rescue or the need of respiratory control during the evacuation from the cold slope site, a considerable number of patients need to receive artificial airway establishment and management, so as to maintain ventilation and further basic and advanced life support. During skiing rescue, the harsh natural environments such as low temperature and steep slope will greatly increase the difficulty of artificial airway establishment and management. To date, there has been no research focusing on the establishment of artificial airway during skiing rescue site. This study aims to simulate the real-world scenario, calculating and comparing the operation time of different artificial airways on the cold slope, and to explore the optimal method of establishing artificial airway on the cold slope, sharing our experience, technical notes, and pitfalls we encountered, hoping to help establish a standard operating procedure in advanced airway management on the ski slope.

## 2. Methods

1. Ethics approval: this research did not involve human subjects; ethics approval is waived.
2. Location: the experiment and data collection were completed in the lower section of Zhonghua Dragon piste (black piste, average steepness 18° and maximum steepness 26°), Wanlong Ski Resort, Chongli District, Zhangjiakou City, Hebei Province, China.
3. Materials: Laerdal Airway Management Trainer (Laerdal Medical, Stavanger, Norway) was used as the stimulator for artificial airway establishment. UE TDC-K3 video laryngoscope (UE Medical Corp., Zhejiang, China), Shiley^™^ TaperGuard oral/nasal tracheal tube (size: 7.5, Medtronic, MN, USA), LMA^®^ Supreme^™^ airway (size: 3, Teleflex Medical Europe Ltd., Westmeath, Ireland), and I-gel^®^ supraglottic airway (size: 3, Intersurgical Ltd., Wokingham, UK) were used for artificial airway establishment.
4. Participant: the artificial airway establishment was completed by an anesthesiologist with more than 10 years of practice experience, wearing full set of ski gears, including the ski suit, helmet, goggles, gloves, and ski boots.
5. Protocol: a 5 ∗ 5 m area was cleared and secured. The simulator was placed inside the area, at the supine position, with the head towards the downhill direction. The participant tried to establish artificial airway, respectively, by endotracheal intubation assisted with the video laryngoscope (endotracheal intubation group), LMA Supreme laryngeal mask insertion (LMA group), and I-gel laryngeal mask insertion (I-gel group). Each procedure was repeated five times. Times of attempts and the completing time (from unwrap of the tracheal tube/laryngeal mask until the artificial airway is established and firmly fixed, ready for further treatment) were recorded.
6. Data analysis: all statistical analyses were performed using SPSS (version 26.0, IBM, Armonk, NY, the United States). Continuous variables were summarized with descriptive statistics (mean and standard deviation). Multiple comparisons were performed using ANOVA, and Tukey's post hoc test was used in comparison between two groups. A *p* value less than 0.05 was considered significant.

## 3. Result

On the day of the test, the temperature of Wanlong Ski Resort was −13°C, with wind speed of 31–51 km/h. In all groups, the artificial airway was successfully deployed in one attempt. The average completion time was 209.2 ± 32.7 seconds in the endotracheal intubation group, 72.2 ± 3.1 seconds in the LMA group, and 52.6 ± 4.2 seconds in the I-gel group. One-way ANOVA showed that there was a significant difference in the completion time among the three groups, *p* < 0.001 (Table 1).

## 4. Discussion

For the first time, this study provides the method and time of establishing artificial airway on a cold slope during the skiing rescue scenario.

Skiing depends on gravity and slope as the driving force, which determines its high-speed nature, related to the high risk of high-energy trauma. In the world's top events such as the FIS (International Ski Federation) World Cup Racing or the Winter Olympic Games, the number of injuries can be as high as 36.7 per 100 athletes per season [3]. In the data released after the 2010–2018 Winter Olympic Games [4–6], skiing and snowboard were the projects with the largest number of people who encountered severe injury, including severe craniocerebral injury and even death [7]. Tourists may also experience cardiac arrest or other serious diseases at high altitude and cold environment. Viglino et al. [2], relying on the emergency network in the Northern Alps of France, analyzed that the cardiac arrest occurred on the slopes of ski resorts in the past 10 years. The results showed that 473 patients had cardiac arrest within the ski resorts, and 136 of them occurred on the slopes of ski resorts. In other two studies focusing on the death cases in snowy mountains in Austria, in 5 and 10 years, the mortality is 0.79% and 0.70%, respectively. The nontraumatic causes of death were mainly cardiac arrest on the slope. The traumatic causes included falling during skiing, collision with other objects or others, and avalanches [1, 8]. In these scenes, emergency airway management and establishment are often required to provide basic life support during on-site skiing rescue and first aid. Thus, it is difficult to control ventilation and protect the airway relying solely on the mask during evacuation from the slope, whether by a rescue toboggan or helicopter. It is also crucial to establish an artificial airway for further treatment. Report showed that, during FIS World Cup Racing in Germany in 2011, at least four anesthesiologists with the first-aid kit, including airway management tools, were on duty at the field of play (FOP) on every competition day. And one of their important responsibilities was to quickly complete the establishment of artificial airway [9]. However, time and methods of establishing artificial airway during skiing rescue have not been reported and not standardized yet.

According to previous studies, when anesthesiologists faced patients with general anesthesia in the operating room, the time to complete endotracheal intubation was about 84 seconds [10], the time to complete LMA Supreme laryngeal mask implantation was about 27 seconds, and the time to complete I-gel laryngeal mask implantation was about 19 seconds [11], which were shorter than those in this study. The time in the operating room comes from patients after general anesthesia, while the time of this study comes from the simulated human. Different objects may cause differences in the establishment time of artificial airway. When the anesthesiologists establish artificial airway in the operating room, the patient lies flat on the operating bed, and the doctor completes the operation in the standing position, within a warm windless circumstance. In this study, the author needs to prone on the ground, with an average gradient of 18 degrees and the maximum gradient of 26 degrees, to complete operations while wearing a full set of ski equipment. The cold weather and strong wind on the test day also had a great impact on the dexterity of the hands. When the operator took off the gloves, the sensory and motor function of the fingers were quickly affected by the low temperature and wind-cold effect. These may be the important reasons why the establishment time of artificial airway during skiing rescue is longer than that in the operating room. The least time-consuming methods are not only favorable for the patients' safety but also prevent the operator from frostbite, avoiding turnovers caused by the clumsiness of the hands.

Among the three methods of artificial airway establishment, endotracheal intubation assisted by using the video laryngoscope is the most complex procedure which requires the most items and steps. And it takes significantly longer time than the other two groups with the laryngeal mask. However, endotracheal intubation is the most accurate and definite way of subglottic ventilation. The possibility of displacement, prolapse, and failure of ventilation is also the lowest. Laryngeal mask is a kind of supraglottic ventilation tool. It does not need special tools (such as the laryngoscope and other endotracheal intubation tools) and can be placed without direct vision. It has lower difficulty and fewer steps. Therefore, the operation time of placing the laryngeal mask is shorter than that of endotracheal intubation. LMA also has the advantages of low training difficulty and simplicity by nonmedical professionals. Based on these advantages, laryngeal mask has been listed as the preferred tool for emergency airway management by the American Society of Anesthesiologists (ASA) [12]. In the comparison of two different types of laryngeal mask, LMA Supreme needs air extraction before use, and the hood needs to be inflated after placement. Therefore, LMA Supreme is slightly more time-consuming than I-gel due to more steps and item requirement, but with no statistical significance.

We also noted that when the average operation time of the endotracheal intubation group was 209 seconds, the standard deviation reached 32 seconds, while the standard deviation of the LMA group and I-gel group was only 3.1 seconds and 4.2 seconds, suggesting that the operation time of the LMA group was relatively close, and the difference was small. The time difference of each operation in the endotracheal intubation group is large, which may be related to the most complex and most steps of endotracheal intubation. This also suggests that, during skiing rescue on the cold slope, the changes of objective conditions such as temperature, slope, wind force, and even the position of the injured, may have a great impact on the establishment of artificial airway. The simpler the operation is, the more the reliability to deal with various conditions and the faster to establish artificial airway.

We want to summarize and share the problems, difficulties, and potential risks encountered by the rescuer during the simulation of advanced airway management on icy slopes. (1) The risk of slippery: when performing advanced airway management on icy slopes, the low friction and the existence of the slope facilitate the occurrence of slippery, not only the rescuers themselves but also the equipments, even the simulator (or actual patients). To the injured patients, slippery might cause secondary damage. To the rescuer, slippery would bring potential risk to both rescuers themselves and also to the injured. Also, on the ski slope, climbing uphill is both energy- and time-consuming, delaying the start of resuscitation. In the endotracheal intubation group, the first attempt and third attempt cost at least 30 seconds more than other attempts in the same group, caused by slippery of the participant (first attempt) and simulator (third attempt). On the ski slope, the rescuer could complete the insertion of the laryngeal mask in the kneeling position, while endotracheal intubation required prone position, which would increase the risk of slippery. Besides, the equipment is hard to get resupply once dropped and drifted away, directly influencing the outcome. The minimal requirement of the endotracheal intubation group includes video laryngoscope, stylet, syringe, and bite block, while the LMA laryngeal mask only needs a syringe for inflation, and I-gel does not require an extra instrument. More tools increase the risk of dropping and drifting. In the fifth attempt, the bite block was dropped and lost; in the real nonmuscle relaxant scenario, missing of the bite block can cause tube biting, resulting in airway obstruction. (2) Difficulty by the position of the rescuer: the insertion of the laryngeal mask does not require direct vision. Even the simulator is placed with the head towards the downhill direction, and the laryngeal mask could be deployed successfully in the kneeling position. Limited difficulty is increased compared to in the operating room. The slope increased the difficulty dramatically by endotracheal intubation. Though, under the navigation of the video laryngoscope, the coincidence of the visual axis, oral axis, pharyngeal axis, and laryngeal axis is not mandatory. On slope conditions, especially steep ones, endotracheal intubation in the kneeling position is nearly impossible even with the guidance of the vision laryngoscope, demanding prone position. In steep slopes, it would cause even more time than intubation for rescuers to find a stable and safe place and position. These are the factors why the endotracheal intubation group is more time-consuming than the other two groups.

### 4.1. Limitations

As an exploratory study, the number of cases is relatively small. The reason is that, as the test repeated, the operator has more experience and adjustment ability. This advance of learning curve will cause bias of operation time. In this study, only the simulated human is operated, and the operation time in the actual human body needs to be further studied in the future. The operator of this study is an anesthesiologist with more than 10 years of clinical experience in artificial airway establishment and management. He has also received special snow sport rescue training program and has relevant experience. For those with less experienced, the data of artificial airway establishment are unknown and need to be further observed. Meanwhile, this research is focusing on calculating the exact time of the operations. Through the simulation, this study aims to analyze and summarize key points, difficulties, and pitfalls of airway management in this specific condition and provide evidence for the real-world scenario.

## 5. Conclusion

The establishment of the artificial airway can be completed by endotracheal intubation assisted by the video laryngoscope and LMA and I-gel laryngeal mask insertion during skiing rescue. And the time of establishing artificial airway by the I-gel laryngeal mask is the shortest in this study. Extra caution should be paid to slippery and drifting.

## Figures and Tables

**Table 1:** Operation time of each group.

Groups	Time (seconds)					
	1st time	2nd time	3rd time	4th time	5th time	Average
Endotracheal intubation group	251	168	232	192	203^*∗∗*^	209.2 ± 32.7^*∗*^
LMA group	73	72	67	74	75	72.2 ± 3.1^*∗*^
I-gel group	59	54	49	52	49	52.6 ± 4.2^*∗*^

^*∗*^ *p* < 0.001. ^*∗∗*^The bite block dropped and drifted away. Tukey's post hoc test showed that the operation time of the endotracheal intubation group was significantly longer than that of the LMA group (209.2 ± 32.7 seconds vs. 72.2 ± 3.1 seconds, *p* < 0.001) and I-gel group (209.2 ± 32.7 seconds vs. 52.6 ± 4.2 seconds, *p* < 0.001). The operation time of the LMA group was 72.2 ± 3.1 seconds, which was not significantly longer than that of the I-gel group (52.6 ± 4.2 seconds, *p*=0.275).

## Data Availability

The datasets used and/or analyzed during the current study are available from the corresponding author upon reasonable request.

## Abbreviations

LMA: Laryngeal mask airway
FIS: International Ski Federation
FOP: Field of play
ASA: American Society of Anesthesiologists.
